# Positive correlation of serum adipocyte fatty acid binding protein levels with carotid–femoral pulse wave velocity in geriatric population

**DOI:** 10.1186/s12877-015-0089-x

**Published:** 2015-07-24

**Authors:** Jen-Pi Tsai, Ji-Hung Wang, Chung-Jen Lee, Yu-Chih Chen, Bang-Gee Hsu

**Affiliations:** Division of Nephrology, Buddhist Tzu Chi General Hospital, Dalin Branch, Chia-Yi, Taiwan; School of Medicine, Tzu Chi University, Hualien, Taiwan; Division of Cardiology, Buddhist Tzu Chi General Hospital, Hualien, Taiwan; Department of Nursing, Tzu Chi College of Technology, Hualien, Taiwan; Division of Nephrology, Buddhist Tzu Chi General Hospital, No. 707, Section 3, Chung-Yang Road, Hualien, 97002 Taiwan

**Keywords:** Adipocyte fatty acid binding protein, Arterial stiffness, Geriatric adults, Pulse wave velocity

## Abstract

**Background:**

Adipocyte fatty acid binding protein (A-FABP) is a novel fat-derived circulating protein, which is independently and positively associated with atherosclerosis. The present study evaluated the relationship between fasting serum A-FABP and central arterial stiffness in geriatric adults.

**Methods:**

Fasting blood samples were obtained from 87 geriatric patients and the serum A-FABP levels were measured using an enzyme immunoassay. Carotid-femoral pulse wave velocity (cfPWV) was determined using the SphygmoCor system. cfPWV values of >10 m/s represented the high arterial stiffness group, while values ≤10 m/s defined the low arterial stiffness group.

**Results:**

High arterial stiffness group comprised of 42 geriatric adults (48.3 %). When compared to those in the low arterial stiffness group, the high arterial stiffness group had a higher rate of diabetes mellitus (*P* = 0.044) and hypertension (*P* = 0.043). Body weight (*P* = 0.027), waist circumference (*P* = 0.035), body mass index (*P* = 0.001), systolic blood pressure (*P* = 0.005), diastolic blood pressure (*P* = 0.045), pulse pressure (*P* = 0.038), and serum A-FABP level (*P* < 0.001) were also higher in the high arterial stiffness group than in the low arterial stiffness group. Multivariate logistic regression analysis of the factors significantly associated with arterial stiffness revealed that A-FABP (odds ratio: 1.833, 95 % confidence interval 1.123–2.993, *P* = 0.015) was an independent predictor of arterial stiffness in geriatric adults.

**Conclusions:**

Serum A-FABP levels constitute a major risk factor in the development of central arterial stiffness in the geriatric population.

## Background

Vessel stiffness is one of the multiple characteristics of cardiovascular (CV) disease, including abnormalities like, the thickening of the vessel wall, formation of fatty streaks, atherosclerotic plaques, and coronary artery calcification [1]. Arterial stiffness is being increasingly recognized as a surrogate end point for CV diseases [2]. The mechanism of arterial stiffness most often involves, degradation of elastin fibers, collagen accumulation, re-organization of cellular elements, and low-grade inflammation with increased expression of tumor necrosis factor-alpha, interleukin-6 and high-sensitivity C-reactive protein [3, 4]. Arterial stiffness reflects sub-clinical organ damage and is considered an important predictor of CV disease. The carotid-femoral pulse wave velocity (cfPWV) is a recommended measure to determine arterial stiffness [5]. A systematic review showed that aortic PWV is a strong predictor of future CV events and all-cause mortality, and has a predictive value independent of classic potential CV risk factors [2].

Aberrant production of the adipokines, due to adiposopathy (fat dysfunction), is an important risk factor for the development of obesity-related CV diseases. Adipocyte fatty acid binding protein (A-FABP), a polypeptide with 132 amino acids and a molecular mass of 14.6 kDa, is one of the most abundant proteins in mature adipocytes, accounting for about 6 % of their total cellular protein content [6]. A-FABP mRNA and protein production are elevated in subcutaneous adipose tissue, as compared to the visceral depot in lean and obese individuals [7]. A-FABP overproduction is believed to play a central role in obesity-related CV disease and endothelial dysfunction by increasing cholesterol and triglyceride accumulation, leading to foam cell development and induction of pro-inflammatory genes. This will in turn promote insulin resistance, atherosclerosis, and potentiate lipid-induced impairment in the activation of endothelial nitric oxide synthase (eNOS) [8].

Previously, we had reported that A-FABP was associated with metabolic syndrome in patients with coronary artery disease and in hemodialysis patients [9, 10]. In addition, arterial stiffness was reported to increase with age. The low prevalence of atherosclerosis in East Asian populations, suggests that the stiffness of arteries was an inevitable consequence of aging [11]. Since CV diseases are a major cause of mortality in the geriatric population, with adipokines playing an important role, we conducted this study to exclude the influences of aging on arterial stiffness. The present study examined the various risk factors in the development of arterial stiffness and evaluated the correlation between the serum concentration of A-FABP and arterial stiffness in geriatric adults.

## Methods

### Participants

Eighty seven elderly volunteers, aged 65 years or older, were enrolled into this study between January and December 2012, at a medical center in Hualien, eastern Taiwan. Trained staff measured blood pressure (BP) in the morning for all participants, using standard mercury sphygmomanometers with appropriate cuff sizes, after the participants had been sitting for at least 10 min. Systolic blood pressure (SBP) and diastolic blood pressure (DBP) were taken at points of appearance and disappearance of the Korotkoff sounds, respectively. SBP and DBP were taken three times at 5-min intervals and were averaged for analysis. Pulse pressure was calculated by subtracting DBP from SBP. In the prevalence survey, hypertension (HTN) was defined as SBP ≥140 mmHg, and/or DBP ≥90 mmHg, or prescription of antihypertensive medication in the past 2 weeks. A person was regarded as having diabetes mellitus (DM), if the fasting plasma glucose was either 126 mg/dL or more, or if he/she was using diabetes medication (oral or insulin) [12]. The current study was approved by the Institutional Review Board of Tzu-Chi University and Hospital. Participants were excluded from the study, if they had an acute infection, acute myocardial infarction, or pulmonary edema at the time of blood sampling, or in case of any reported use of calcium, active vitamin D metabolites, bisphosphonates, teriparatide, or estrogens medication, or if they declined to provide written informed consents for the study.

### Anthropometric analysis

Body weight was measured in patients wearing light clothing and without shoes, to the nearest 0.5 kilograms, and height was measured to the nearest 0.5 cm. Waist circumference was measured by tape around the patient’s waist, from the point of the lowest ribs to the hip bones by placing patient’s hands on hips. Body mass index (BMI) was calculated as the weight in kilograms divided by the height in meters squared [13].

### Biochemical investigations

Fasting blood samples (approximately 5 mL) were collected and immediately centrifuged at 3000 *g* for 10 min. Serum levels of blood urea nitrogen (BUN), creatinine (Cre), fasting glucose, total cholesterol (TCH), triglycerides (TG), high-density lipoprotein cholesterol (HDL-cholesterol), low-density lipoprotein cholesterol (LDL-cholesterol), total calcium, and phosphorus, were measured using an autoanalyzer (COBAS Integra 800, Roche Diagnostics, Basel, Switzerland) [13–15]. Serum A-FABP levels were measured using a commercially available enzyme immunoassay (EIA; SPI- BIO, Montigny le Bretonneux, France) [9, 10]. Serum intact parathyroid hormone levels (iPTH) were measured using enzyme-linked immunosorbent assays (ELISA; Diagnostic Systems Laboratories, Webster, Texas, USA) [15]. The estimate glomerular filtration rate (GFR) was calculated in this study using the Modification of Diet in Renal Disease (MDRD) equation.

### Carotid-femoral pulse wave velocity (cfPWV) measurements

The cfPWV were measured using a pressure tonometer (SphygmoCor system, AtCor Medical, Australia), and the pressure pulse waveform in the underlying artery was recorded transcutaneously, as previously described [14, 15]. All measurements were taken in the morning while the participants are in a supine position, after a minimum of 10-min rest in a quiet, temperature-controlled room. Records were made simultaneously with an ECG signal, which provided an *R*-timing reference. Pulse wave recordings were performed consecutively at two superficial artery sites (carotid-femoral segment). Integral software was used to process each set of the pulse wave and ECG data, to calculate the mean time difference between the *R*-wave and the pulse wave on a beat-to-beat basis, with an average of ten consecutive cardiac cycles. The cfPWV was calculated using the distance and the mean time difference between the two recorded points. Quality indices, included in the software, were set to ensure the uniformity of data. In this study, cfPWV values of >10 m/s were used to define the high arterial stiffness group, while values ≤10 m/s were regarded as the low arterial stiffness group, according to the ESH-ESC 2013 Guidelines [5].

### Statistical analysis

Data are expressed as the mean ± standard deviation (SD) and were tested for normal distribution using Kolmogorov-Smirnov statistics. Comparisons between patients were performed using the Student’s independent *t*-test (2-tailed) for normally distributed data, or the Mann–Whitney *U* test for parameters that presented a non-normal distribution (TG, fasting glucose, and iPTH). Data expressed as the number of patients were analyzed by the *χ*^2^ test. Variables that were significantly associated with arterial stiffness in geriatric adults were tested for independence by multivariate logistic regression analysis (adapted factors: DM, HTN, body weight, waist circumference, BMI, SBP, DBP, pulse pressure, and A-FABP). Data were analyzed using SPSS for Windows (version 19.0; SPSS Inc., Chicago, IL, USA). A *P*-value < 0.05 was considered statistically significant.

## Results

Demographic, biochemical, and clinical characteristics of the 87 geriatric adults are shown in Tables 1 and 2. The medical histories of the geriatric adults included DM (*n* = 34 [39.1 %]), and HTN (*n* = 42 [48.3 %]). The medications prescribed to the geriatric adults included angiotensin-converting enzyme inhibitors (ACEi; *n* = 13 [14.9 %]), angiotensin receptor blockers (ARB; *n* = 30 [34.5 %]), β-blockers (*n* = 24 [27.6 %]), calcium channel blockers (CCB; *n* = 22 [25.3 %]), statins (*n* = 28 [32.2 %]), and fibrate (*n* = 10 [11.5 %]). Forty-two geriatric adults (48.3 %) belonged to the high arterial stiffness group, and when compared to the geriatric adults in the low arterial stiffness group, they had a higher incidence of DM (31.1 % vs. 52.4 %, *P* = 0.044) or HTN (37.8 % vs. 59.5 %, *P* = 0.043). There was no statistically significant difference between the high and low arterial stiffness groups, based on, gender, ACEi, ARB, β-blocker, CCB, statins, or fibrate use.

**Table 1:** Clinical and analytical characteristics of 87 geriatric adults

Items	Parameter		Parameter	
Anthropometric findings	Height (cm)	160.08 ± 7.81	Waist circumference (cm)	91.26 ± 10.81
	Body weight (kg)	64.80 ± 10.54		72.18 ± 5.36
	Body mass index (kg/m^2^)	25.29 ± 3.74	Age (year)	131.86 ± 17.96
	DBP (mmHg)	71.33 ± 8.71	SBP (mmHg)	60.53 ± 15.78
			Pulse pressure (mmHg)	
Biochemical findings	Triglyceride (mg/dL)	133.87 ± 78.34	Total cholesterol (mg/dL)	174.44 ± 36.56
	Fasting glucose (mg/dL)	126.30 ± 44.67	HDL-cholesterol (mg/dL)	47.28 ± 12.74
	LDL-cholesterol (mg/dL)	103.84 ± 34.33	Creatinine (mg/dL)	1.19 ± 0.37
	BUN (mg/dL)	17.97 ± 6.31	GFR (mL/min)	64.53 ± 20.17
	Total calcium (mg/dL)	9.13 ± 0.38	Phosphorus (mg/dL)	3.44 ± 0.38
	Ca × P product (mg^2^/dL^2^)	31.39 ± 4.46	iPTH (pg/mL)	58.31 ± 34.53
	A-FABP (ng/mL)	22.73 ± 13.46	cfPWV (m/s)	10.13 ± 2.92

Data are expressed as means ± standard deviations

*SBP* systolic blood pressure, *DBP* diastolic blood pressure, *HDL-cholesterol* high-density lipoprotein cholesterol, *LDL-cholesterol* low-density lipoprotein cholesterol, *BUN* blood urea nitrogen, *GFR* glomerular filtration rate, *Ca × P product* calcium–phosphorus product, *iPTH* intact parathyroid hormone, *A-FABP* adipocyte fatty acid binding protein, *cfPWV* carotid-femoral pulse wave velocity

**Table 2:** Clinical characteristics and carotid-femoral pulse wave velocity levels of 87 geriatric adults

Characteristic		Low AS group (%)	High AS group (%)	*P* value
Gender	Male	31 (68.9)	29 (69.0)	0.897
	Female	14 (31.1)	13 (31.0)	
Diabetes	No	31 (68.9)	20 (47.6)	0.044*
	Yes	14 (31.1)	22 (52.4)	
Hypertension	No	28 (62.2)	17 (40.5)	0.043*
	Yes	17 (37.8)	25 (59.5)	
ACE inhibitor	No	41 (91.1)	35 (83.3)	0.275
	Yes	4 (8.9)	7 (16.7)	
ARB	No	33 (73.3)	24 (57.1)	0.112
	Yes	12 (26.7)	18 (42.9)	
β-blocker	No	35 (77.8)	28 (66.7)	0.247
	Yes	10 (22.2)	14 (33.3)	
CCB	No	36 (80.0)	29 (69.0)	0.240
	Yes	9 (20.0)	13 (31.0)	
Statins	No	29 (64.4)	30 (71.4)	0.486
	Yes	16 (35.6)	12 (28.6)	
Fibrate	No	40 (88.9)	37 (88.18)	0.908
	Yes	5 (11.1)	5 (11.9)	

Data are expressed as number of patients and analysis was done using the *χ* ^2^ test

*AS* arterial stiffness, *cfPWV* carotid-femoral pulse wave velocity, *ACE* angiotensin-converting enzyme, *ARB* angiotensin-receptor blocker, *CCB* calcium-channel blocker

*P < 0.05 was considered statistically significant after Student *t*-test

Body weight (67.37 ± 10.91 kg vs. 62.40 ± 9.69 kg, *P* = 0.027), waist circumference (93.79 ± 11.99 cm vs. 88.91 ± 9.10 cm, *P* = 0.035), BMI (26.63 ± 3.83 kg/m^2^vs. 24.04 ± 3.21 kg/m^2^, *P* = 0.001), SBP (137.40 ± 18.68 mmHg vs. 126.69 ± 15.77 mmHg, *P* = 0.005), DBP (73.26 ± 7.71 mmHg vs. 69.53 ± 9.27 mmHg, *P* = 0.045), pulse pressure (64.14 ± 16.54 mmHg vs. 57.16 ± 14.42 mmHg, *P* = 0.038) were higher in the high arterial stiffness group, as compared to the low arterial stiffness group. Moreover, serum A-FABP was also elevated in the high arterial stiffness group than in the low arterial stiffness group (28.13 ± 13.86 ng/ml vs. 17.69 ± 11.01 ng/ml, *P* < 0.001; Table 3).

**Table 3:** Clinical variables of the 87 geriatric adults with or without arterial stiffness

Items	Low AS group (*n* = 45)	High AS group (*n* = 42)	*P* value
Age (years)^a^	71.71 ± 5.36	72.69 ± 5.38	0.397
Height (cm)^a^	161.02 ± 7.54	159.07 ± 8.04	0.246
Body weight (kg)^a^	62.40 ± 9.69	67.37 ± 10.91	0.027*
Waist circumference (cm)^a^	88.91 ± 9.10	93.79 ± 11.99	0.035*
Body mass index (kg/m^2^)^a^	24.04 ± 3.21	26.63 ± 3.83	0.001*
cfPWV (m/s)^a^	7.89 ± 1.20	12.50 ± 2.56	<0.001*
SBP (mmHg)^a^	126.69 ± 15.77	137.40 ± 18.68	0.005*
DBP (mmHg)^a^	69.53 ± 9.27	73.26 ± 7.71	0.045*
Pulse pressure (mmHg)^a^	57.16 ± 14.42	64.14 ± 16.54	0.038*
Total cholesterol (mg/dL)^a^	174.69 ± 31.80	174.17 ± 41.45	0.947
Triglyceride (mg/dL)^b^	131.60 ± 83.17	136.31 ± 73.74	0.405
HDL-C (mg/dL)^a^	49.60 ± 12.87	44.78 ± 12.27	0.078
LDL-C (mg/dL)^a^	102.89 ± 27.96	104.86 ± 40.38	0.791
Fasting glucose (mg/dL)^b^	123.73 ± 47.51	129.05 ± 41.82	0.271
Blood urea nitrogen (mg/dL)^a^	16.76 ± 5.23	19.26 ± 7.13	0.064
Creatinine (mg/dL)^a^	1.13 ± 0.33	1.26 ± 0.41	0.108
GFR (mL/min)^a^	67.67 ± 19.18	61.17 ± 20.87	0.134
Total calcium (mg/dL)	9.16 ± 0.40	9.09 ± 0.37	0.363
Phosphorus (mg/dL)	3.43 ± 0.48	3.46 ± 0.46	0.782
Ca × P product (mg^2^/dL^2^)	31.40 ± 4.39	31.43 ± 4.60	0.941
iPTH (pg/mL)^b^	56.44 ± 36.99	60.32 ± 32.01	0.165
A-FABP (ng/mL)^a^	17.69 ± 11.01	28.13 ± 13.86	<0.001*

*AS* arterial stiffness, *cfPWV* carotid-femoral pulse wave velocity, *SBP* systolic blood pressure, *DBP* diastolic blood pressure, *HDL-C* high density lipoprotein-cholesterol, *LDL-C* low density lipoprotein-cholesterol, *GFR* glomerular filtration rate, *Ca × P product* calcium–phosphorus product, *iPTH* intact parathyroid hormone, *A-FABP* adipocyte fatty acid binding protein

*P < 0.05 was considered statistically significant after the Student’s *t*-test or Mann–Whitney *U* test

^a^data were tested using the Student’s *t*-test

^b^data were tested using the Mann–Whitney *U* test

Multivariate logistic regression analysis of the factors significantly associated with arterial stiffness (DM, HTN, body weight, waist circumference, BMI, SBP, DBP, pulse pressure, and A-FABP) revealed that A-FABP (odds ratio: 1.833, 95 % confidence interval (CI): 1.123–2.993, *P* = 0.015) was an independent predictor of arterial stiffness in geriatric adults (Table 4).

**Table 4:** Correlation between arterial stiffness among the 87 geriatric adults by multivariate logistic regression analysis

Variables	Odds ratio	95 % CI	*P* value
A-FABP (ng/mL) (each increase of 1 ng/mL)	1.833	1.123–2.993	0.015*
Diabetes	1.737	0.621–4.858	0.293
Hypertension	1.360	0.465–3.982	0.575
Body weight (kg) (each increase of 1 kg)	1.049	0.949–1.160	0.351
Waist circumference (cm) (each decrease of 1 cm)	0.970	0.895–1.052	0.463
BMI (kg/m^2^) (each increase of 1 kg/m^2^)	1.124	0.858–1.472	0.397
SBP (mmHg) (each increase of 1 mmHg)	1.022	0.988–1.057	0.207
DBP (mmHg) (each increase of 1 mmHg)	1.023	0.956–1.094	0.514

*CI* confidence Interval, *A-FABP* adipocyte fatty acid binding protein, *BMI* body mass index, *SBP* systolic blood pressure, *DBP* diastolic blood pressure

*P < 0.05 was considered statistically significant in the multivariate logistic regression analysis (adopted factors: diabetes, hypertension, body weight, waist circumference, BMI, SBP, DBP, pulse pressure and A-FABP)

## Discussion

The current results reveal that the fasting A-FABP levels were higher in the high arterial stiffness group than in the low arterial stiffness group, and A-FABP is an independent predictor for the development of arterial stiffness in the geriatric population.

A systematic review had shown that BP was independently associated with PWV in approximately 90 % of the studies [16]. Cecelja et al. found a dissociation of cfPWV with classic factors, such as gender, smoke, or lipids other than HTN, and they suggested that aortic stiffing might be driven by an alternative pathology, which depended on mechanical stretch of the arterial wall [16]. The pathophysiological correlation between arterial stiffness and HTN is that the increased arterial stiffness reduces the lumen diameter, for a given smooth muscle tone and blood pressure, leading to a premature return of the reflected wave in late systole, thereby increasing the central pulse pressure and SBP, and decreasing DBP [17]. On the other hand, aortic stiffness may affect aortic function, reduce baroreceptor responsiveness, and increase systolic pressure, making arterial stiffness one of the leading causes of increased blood pressure [4]. Meta-analysis of studies in the field have demonstrated that an increase in aortic PWV by 1 m/s corresponded to an age-, sex-, and risk factor-adjusted increase in total CV events, CV mortality, and all-cause mortality by 14, 15, and 15 %, respectively [2]. In addition, another meta-analysis found that greater arterial stiffness correlated with cerebral small vessel diseases [18], and Zhang et al. supposed that measuring cfPWV was beneficial to the elderly regarding the cardiovascular and cerebrovascular diseases, including atherosclerosis, cognitive dysfunction and mortality [19]. Therefore, arterial stiffness could serve as an independent predictor of coronary events and adverse CV diseases.

Studies had shown that there is a crosstalk between CV diseases and adipokines, such as, adiponectin, leptin, and A-FABP [20–23]. Makowski and colleagues reported that apolipoprotein E deficient mice with A-FABP +/+ adipocytes and A-FABP −/− macrophages, showed a comparable reduction in atherosclerotic lesions to those with total A-FABP deficiency, indicating an independent role for macrophage A-FABP in atherogenesis [24]. Lee et al. had shown an enhanced expression of A-FABP on aortic endothelium of apolipoprotein E deficient mice and in cultured human microvascular endothelial cells. This lipid-induced A-FABP expression was associated with reduced phosphorylated eNOS and NO production, and was reversed by A-FABP inhibitor [8]. So, the pro-atherogenic potential of A-FABP on the vasculature is possibly mediated by the pro-inflammatory effects, independent of lipid metabolism and insulin sensitivity. Further, it contributes to the endothelial dysfunction by potentiating lipid-induced impairment in eNOS activation, and thus endothelium-dependent vasodilatation.

Clinical reports reveal a significant correlation of baseline serum A-FABP with BMI, homeostasis model of insulin resistance, and cardio-ankle vascular index, which is a marker of arterial stiffness [25, 26]. In addition to the associated metabolic risk factors, A-FABP levels were positively correlated with coronary plaque volume burden as well, and may serve as a biomarker for the detection of coronary artery disease [22, 23]. An increase in the number of stenotic coronary arteries resulted in a corresponding increase in the plasma A-FABP levels in patients with coronary heart diseases [23]. In addition to the macrovascular complications (ischemic heart disease, stroke, or peripheral vascular disease) in diabetic patients, A-FABP show a positive correlation with albuminuria and negative correlation with glomerular filtration rate, indicating that the serum level of A-FABP is influenced by impaired renal clearance and activated macrophages in diabetic nephropathy [27]. In this study, we found that geriatric adults who had high arterial stiffness would have higher serum A-FABP levels than those with low arterial stiffness. Moreover, A-FABP was demonstrated to be an independent risk factor for developing high arterial stiffness.

Classic CV risk factors, including DM, hyperlipidemia, elevated BMI and smoking, had been implicated in accelerating arterial stiffness. However, Cecelja et al. have indicated that the prognostic value of cfPWV may be related to a process of arterial ageing, and probably unrelated to the classic risk factors, other than HTN [16]. Additionally, impaired glucose tolerance and DM in a population-based study was independently associated with the central arterial stiffness, after adjusting mean arterial pressure, age and gender [28]. In our geriatric patients, we found that adults who were found to have high arterial stiffness would have a higher body weight, waist circumference, BMI, SBP, DBP, and pulse pressure. Furthermore, those who were in the high arterial stiffness group also had a higher percentage of DM or HTN, as previously reported [16].

Several types of medications had been demonstrated to affect arterial stiffness. Studies of β-blockers on arterial stiffness showed less impact on central BP decline compared with the peripheral BP [29, 30]. Older hypertensive patients treated with calcium channel blockers, showed the lowest central aortic pressure and less augmentation pressure, when compared with the placebo [31]. Moreover, usage of ACEi or ARB showed significant effects on reducing the central BP and augmentation index, through the reduction of oxidative stress and inflammation, and vasodilatation through angiotensin II inhibition [30, 31]. Miyoshi et al. conducted a study which showed that the effects of ARB in HTN patients could reduce cardio-ankle vascular index as well as serum levels of A-FABP, but the causal relationship needed to be clarified by further studies [26]. Even though, one systematic review showed controversial effects of statin on the reduction of aortic PWV [32], recent reports indicated that low dose of atorvastatin in mild HTN and hypercholesterolemia exerted beneficial effects on arterial stiffness and central aortic pressure [33]. In obese patients without glucose intolerance, treatment with PPAR alpha agonist (fenofibrate) revealed significant reduction in the augmentation index, PWV, and pro-inflammatory markers [34]. The results of subgroup analysis in this study suggest that ACEi, ARB, β-blockers, CCB, statins, or fibrate had no influence at all, on arterial stiffness.

## Conclusions

The major limitation of this study is that, this was a cross-sectional study with a limited number of geriatric adults. Therefore, the findings of this study must be confirmed by long-term prospective studies before a causal relationship between the serum A-FABP and arterial stiffness can be established in the geriatric patient population. However, to the best of our knowledge, this is the first study to examine the relationship between serum A-FABP and arterial stiffness in the geriatric population.

In conclusion, we found that serum A-FABP levels were higher in geriatric population having high arterial stiffness. More importantly, A-FABP may serve as an independent predictor for the development of arterial stiffness in geriatric adults. Whether serum A-FABP directly contributes to the development of arterial stiffness or not, needs to be further validated in larger cohorts.

## Abbreviations

A-FABP: Adipocyte fatty acid binding protein
ACEi: Angiotensin-converting enzyme inhibitor
ARB: Angiotensin receptor blocker
BMI: Body mass index
BP: Blood pressure
BUN: Blood urea nitrogen
CCB: Calcium channel blockers
cfPWV: Carotid-femoral pulse wave velocity
DM: Diabetes mellitus
CVD: Cardiovascular disease
Cre: Creatinine
DBP: Diastolic blood pressure
eNOS: Endothelial nitric oxide synthase
GFR: Glomerular filtration rate
HDL-cholesterol: High-density lipoprotein-cholesterol
HTN: Hypertension
iPTH: Intact parathyroid hormone levels
MDRD: Modification of diet in renal disease
LDL-cholesterol: Low-density lipoprotein cholesterol
SBP: Systolic blood pressure
TCH: Total cholesterol
TG: Triglyceride
